# Improving Compliance with Vascular Access Devices Management Standards Using a Multidisciplinary Approach

**DOI:** 10.1017/ash.2025.289

**Published:** 2025-09-24

**Authors:** Denisse Silva, Sherry R. Reid, Michelle L Covington-Bailey, Aderonke Badejogbin, Zanzine Boullt, Matthew Garza, Eduardo J. Herrera, Abimbola Owoyele, Tanisha Robertson, Ikwo Oboho

**Affiliations:** 1 VA North Texas Health Care System, Dallas, Texas; 2 Veterans Affairs North Texas Health Care System; 3 VA North Texas Healthcare System; 4 VA North Texas Health Care System; 5 Dallas VA Medical Center; 6 VA North Texas Health Care System/UT Southwestern University Medical Center

## Abstract

**Background:** During the COVID-19 pandemic, the rate of central line-associated bloodstream infections (CLABSI) decreased at the Veteran Affairs North Texas Health Care System. From fiscal year (FY) 2022 Quarter (Q)4 to FY2023 Q2, the CLABSI rate increased from 0 to 0.79 per 1,000 device days. Breaches in evidence-based practices for the maintenance of vascular access devices (VAD) were hypothesized to have contributed to the increase in CLABSI rate. **Methods:** In March 2023, a multidisciplinary workgroup was created with the primary goal of improving compliance with VAD standards of care to ≥ 95% by FY2023 Q4 and a secondary goal of decreasing CLABSI rates. A baseline assessment of 12 VAD insertion and maintenance process measures was developed using an assessment tool to record nurses’ observations and review documentation in the computerized patient record system. In addition, the facility VAD policy was updated, and nurses received competency training on VAD management. Baseline compliance data for the 12 VAD process measures was compared to data during the intervention period for acute and critical care areas. CLABSI rates (classified using the National Healthcare Safety Network surveillance criteria) were compared to the period before the creation of the workgroup, policy updates, and training. **Results:** Nurse observations in acute and critical care units during FY2023 were 19 (Q2), 1,284 (Q3), and 718 (Q4). From FY2023 Q2 to Q4, three of the 12 process measures met the ≥ 95% compliance goal by FY2023 Q4. The process measures that met the goal from Q2 to Q4 were clean peripheral IV catheter hub: 100% to 95.0%, unused tubing Y-sites capped with swap cap: 0% to 96.0%, and documentation of the last dressing change in CPRS: 0% to 99.0%. Notable increases were also seen for three other measures: appropriately dating of peripheral IV tubing: 78.9% to 88.0%, presence of Coban or kerlix occluding site: 0% to 46.0%, and documentation of device insertion: 0% to 89.0%. Persistent deficits were noted in the documentation of peripheral intravenous dressing dates and initials (compliance **Conclusions:** Enlisting a multidisciplinary team approach, including training, and updating VAD policy/procedures, led to a moderate improvement in VAD management compliance and a decline in CLABSI rates.

